# A VHF-Voltage Double Time Integral Method for Apparent-Charge Estimation of Partial Discharge in Gas-Insulated Switchgear

**DOI:** 10.3390/s26206500

**Published:** 2026-10-14

**Authors:** Subin Gao, Xiaoli Zhou, Chao Pan, Yong Qian, Gehao Sheng

**Affiliations:** 1 College of Intelligent Robotics and Advanced Manufacturing, Fudan University, Shanghai 200433, China; 24210720022@m.fudan.edu.cn; 2 School of Electronic Information and Electrical Engineering, Shanghai Jiao Tong University, Shanghai 200240, China; sjtupc99@sjtu.edu.cn (C.P.); qian_yong@sjtu.edu.cn (Y.Q.); shenghe@sjtu.edu.cn (G.S.)

**Keywords:** partial discharge, apparent-charge measurement, VHF sensor, gas-insulated switchgear, double time integral of voltage

## Abstract

Gas-insulated switchgear (GIS) is widely used in modern power systems, but transportation, installation, and manufacturing defects can trigger partial discharge (PD). Apparent charge is a key indicator of PD intensity and insulation deterioration, yet conventional pulse current measurements perform poorly in field environments because of interference, pulse superposition, non-flat sensor responses, and impedance mismatch. Very-high-frequency (VHF) sensing is sensitive to on-site PD but lacks a direct quantitative mapping to apparent charge. This study derives and optimizes a double time integral of voltage (DTIV) method for capacitively coupled VHF sensors. Simulation and experimental platforms were established, and through-origin linear regression was used to relate DTIV output to reference apparent charge. The first and second voltage zero-crossings (ZC1 and ZC2) were evaluated as integration upper limits, and the preferred DTIV_ZC2 strategy was further assessed using thresholds of 1% and 5% of the signal peak. For the simulated library, using the second zero-crossing increased the coefficient of determination from 0.8957 to 0.9585 and reduced the average relative error to 4.5%. A 5% threshold further improved these values to 0.9641 and 3.9%, respectively. The results support the feasibility of calibrated DTIV estimation for the tested sensor and ideal laboratory configuration.

## 1. Introduction

Partial discharge (PD) measurement is a common insulation-diagnostic technique and has become an essential pre-commissioning test for gas-insulated switchgear (GIS) in field applications [1,2,3]. Apparent charge is a key metric for evaluating PD intensity and insulation deterioration, and it is also the central measurand in PD measurement systems [4].

The pulse current method (PCM) is the standard method for measuring the apparent charge of PD. IEC 60270 [4] specifies the relationship between apparent charge and the time integral of the PD pulse waveform; however, the method assumes a low-interference and impedance-matched laboratory circuit. In field GIS environments, external interference, pulse overlap, non-flat sensor frequency response, and impedance mismatch can distort PD pulse waveforms. Consequently, PCM is difficult to apply directly under on-site operating conditions [5,6,7,8].

For strongly disturbed field environments, non-conventional electromagnetic detection methods are widely used [9,10], while ultrasonic and optical techniques provide complementary approaches [11,12]. Nevertheless, the non-flat frequency response and impedance mismatch of non-conventional sensors distort the output waveform, making it difficult to obtain quantitative results that are consistent with PCM-based apparent charge [13,14,15]. Quantitative charge estimation with electric and magnetic sensors requires calibration appropriate to their response and installation [16].

Considerable efforts have been made to overcome the difficulty of quantifying apparent charge using non-conventional methods. Ohtsuka et al. investigated the relationship between VHF signals and the charge derived from PD current waveforms in a GIS model, although the result was influenced by propagation mode and sensor response [17]. Tao et al. proposed an FDTD-based digital-model method for calculating discharge quantity from the second time integral of UHF signals, which provides a basis for the present study [18]. These studies mainly focused on sensor response or interference identification, while a concrete quantitative derivation between non-conventional sensor output and apparent charge remains insufficient.

The double time integral of voltage (DTIV) method offers a promising route for charge estimation. Rodrigo-Mor et al. applied DTIV to high-frequency current transformer (HFCT) measurements and compared several charge-estimation methods for cable PD measurements, showing that DTIV is applicable to sensors whose transfer functions can be represented by real zero-pole models [19,20]. Rodrigo-Mor subsequently designed a magnetic-loop antenna satisfying the transfer-function requirement and applied DTIV to charge estimation with high accuracy [21,22,23]. Mier et al. further discussed reflection suppression, propagation path, and sensor response from the perspective of synergistic VHF electric and magnetic sensing in GIS [7,24].

The response of a capacitively coupled VHF sensor is governed by its coupling and parasitic capacitances, load impedance, and related circuit parameters. An equivalent-circuit treatment provides the relationship between this response and the voltage double integral [16]. This study develops a calibrated DTIV approach for a capacitively coupled VHF sensor and evaluates the effects of integration-endpoint and amplitude-threshold selection. The approach is examined through comparisons of ZC1 and ZC2 and of 1% and 5% thresholds, followed by controlled waveform and physical artificial-defect experiments on a laboratory GIS platform.

## 2. Charge-Estimation Principle

### 2.1. Charge-Estimation Principle of the Pulse Current Method

IEC 60270 defines the apparent charge Q of a PD event as the time integral of the short-duration PD pulse i_PD_(t) at the test-object terminal, and requires the integral response to be equivalent to the system response produced by the calibration charge Q_0_ under the same measuring circuit. The expression is given as follows:

$$Q=\int_{t_{1}}^{t_{2}}i_{\mathrm{PD}}\left(t\right)\mathrm{dt},$$

where i_PD_(t) is the actual short-duration PD pulse, and t1 and t2 are the lower and upper integration limits, which are commonly set at signal zero crossings.

The calibration charge Q_0_ is obtained by integrating the calibration current i_cal_(t) generated by the calibrator, as expressed below:

$$Q_{0}=\int_{t_{1}}^{t_{2}}i_{\mathrm{cal}}\left(t\right)\mathrm{dt}.$$

On this basis, the standard further specifies the following:

(1) The apparent-charge measuring system shall be equipped with a band-pass filter whose amplitude–frequency flatness is no greater than ±1 dB and whose phase linearity is within ±5°.
(2) The calibration injection unit shall output a unipolar current pulse with a rise time no greater than 5 ns and a half-peak width no greater than 100 ns. The calibration charge shall be traceable to the national charge standard, with an expanded uncertainty no greater than 2%.
(3) The integration window [0, t] shall cover 10–90% of the pulse energy, and the system pulse-time resolution shall be no greater than 100 μs.

According to the above definition and requirements, the apparent charge Q is used as the reference charge. The apparent-charge estimate obtained from the sensor output is compared with this reference to evaluate the accuracy of the proposed charge-estimation method.

In field environments, PD signals are measured by various sensors. The sensor output y(t) can be expressed as:

*y*(*t*) = *i*_PD_(*t*) ∗ *h*(*t*),

where h(t) is the sensor impulse response, and ∗ denotes convolution in the time domain. For causal, absolutely integrable signals observed over the complete response, the total time integral of the convolution is expressed as:

$$\int_{0}^{\infty }y(t)\mathrm{dt}=\left[\int_{0}^{\infty }i_{\mathrm{PD}}\left(t\right)\mathrm{dt}\right]\left[\int_{0}^{\infty }h\left(t\right)\mathrm{dt}\right]=Q\int_{0}^{\infty }h\left(t\right)\mathrm{dt}.$$

Direct integration of a sensor output requires a known relationship between that output and the reference current. Integrating the complete response of an arbitrary band-pass sensor does not generally recover the injected charge. This limitation concerns direct waveform integration and does not invalidate calibrated conventional apparent-charge measurements.

For the illustrative band-pass response considered here, the time integral of the impulse response can be written as follows:

$$\int_{0}^{t}h(t)\mathrm{dt}=\frac{k}{b-a}\left(e^{-\mathrm{at}}-e^{-\mathrm{bt}}\right)\to 0,t\to \infty ,$$

where k is the system gain, and a and b are the attenuation coefficients corresponding to the low- and high-frequency poles, respectively. For this band-pass response, the total impulse-response integral approaches zero as the upper limit tends to infinity. Consequently, the total integral of the measured pulse also approaches zero, and direct first-integration charge estimation fails.

### 2.2. Charge Estimation Based on the Double Time Integral of Voltage

This section derives the DTIV relation from the sensor equivalent circuit and identifies the conditions under which it can be used for charge estimation [16]. In contrast to direct integration of the band-pass output discussed above, the double integral retains a low-frequency scale factor, subject to the circuit, propagation, and integration-window assumptions stated below.

**Figure 1:**
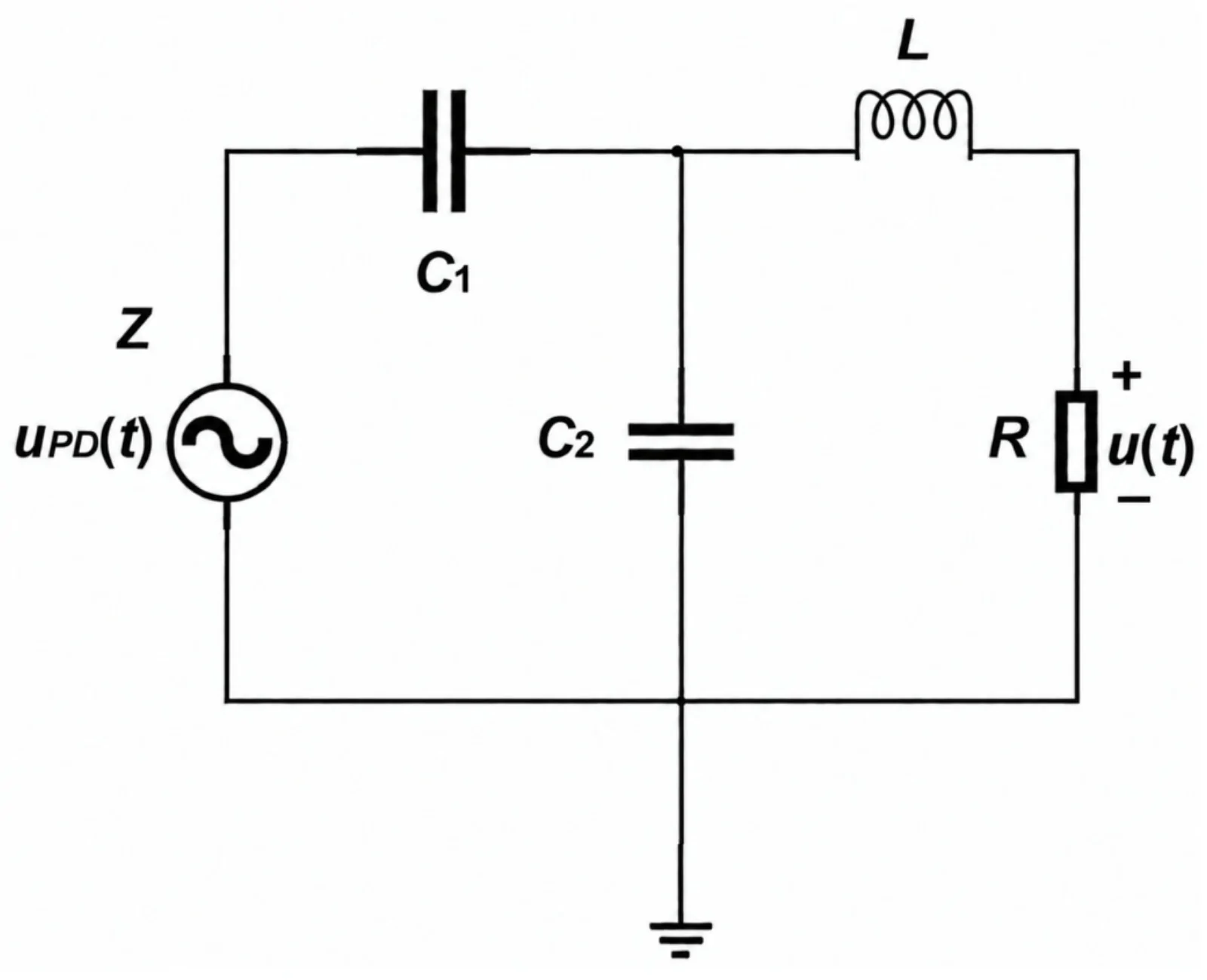
Schematic diagram of the VHF sensor circuit.

A VHF sensor is considered in this section, and its circuit structure can be represented by the resistor–inductor–capacitor (RLC) equivalent circuit shown in Figure 1. The VHF band lies between 30 and 300 MHz. Calculation shows that, for the coaxial GIS structure used in this paper, the cutoff frequency is lower than that of higher-order modes and propagation is dominated approximately by the transverse electromagnetic (TEM) mode; therefore, the assumptions of the proposed algorithm are satisfied.

Let the excitation voltage be *u*_PD_(*t*), coupled through *C*_1_ to the network in Figure 1, and let *u*(*t*) be the output across *R*. Denote the junction voltage by *v*(*t*). The inductor branch gives *v* = *u* + (*L*/*R*)*u*^′^, and current balance gives $C_{1}(u_{\mathrm{PD}}^{\prime }-v^{\prime })=C_{2}v^{\prime }+u/R$. Defining *C*_Σ_ = *C*_1_ + *C*_2_ yields the time-domain equation

$$LC_{\Sigma }\frac{d^{2}u(t)}{dt^{2}}+RC_{\Sigma }\frac{\mathrm{du}(t)}{\mathrm{dt}}+u(t)=RC_{1}\frac{du_{\mathrm{PD}}(t)}{\mathrm{dt}}.$$

The reduced expression with *C*_2_ in the dynamic coefficients follows only when *C*_1_≪*C*_2_; the derivation below retains *C*_Σ_ without requiring that approximation. Take *t* = 0 before pulse arrival and assume the local relationship *u*_PD_(*t*) = *Zi*_PD_(*t*) with constant *Z*. Define *a* = *RZC*_1_ and the following finite-window integrals:

$$Q(T)=\int_{0}^{T}i_{\mathrm{PD}}(t)\mathrm{dt},J(T)=\int_{0}^{T}u(t)\mathrm{dt},$$

$$S(T)=\int_{0}^{T}[\int_{0}^{t}u(\tau )\mathrm{d\tau}]\mathrm{dt}=\int_{0}^{T}(T-t)u(t)\mathrm{dt}.$$

Integration by parts retains the initial-state terms explicitly:

$$\int_{0}^{T}(T-t)u^{\prime \prime }(t)\mathrm{dt}=u(T)-u(0)-Tu^{\prime }(0),$$

$$\int_{0}^{T}(T-t)u^{\prime }(t)\mathrm{dt}=J(T)-\mathrm{Tu}(0),$$

$$\int_{0}^{T}(T-t)i_{\mathrm{PD}}^{\prime }(t)\mathrm{dt}=Q(T)-Ti_{\mathrm{PD}}(0).$$

Applying these identities to the circuit equation gives

*a*[*Q*(*T*) − *Ti*_PD_(0)] = *LC*_Σ_[*u*(*T*) − *u*(0) − *Tu*^′^(0)]

+*RC*_Σ_[*J*(*T*) − *Tu*(0)] + *S*(*T*).

For quiescent initial conditions, *u*(0) = *u*^′^(0) = *i*_PD_(0) = 0, the finite-window relation becomes

*aQ*(*T*) = *LC*_Σ_*u*(*T*) + *RC*_Σ_*J*(*T*) + *S*(*T*).

The proportionality below requires the source pulse to be complete within the window, the voltage response to have sufficiently decayed, and the remaining single integral to be negligible:

$$Q\approx \frac{S(T)}{\mathrm{RZ}C_{1}}\approx \frac{1}{\mathrm{RZ}C_{1}}\iint u\left(t\right)dt^{2}.,Q_{\mathrm{ref}}=\int_{0}^{\infty }i_{\mathrm{PD}}(t)\mathrm{dt}.$$

Under these assumptions, the model charge is approximately proportional to the voltage double integral. In the experiments, its correspondence to reference apparent charge is established by calibration of the measurement chain. The theoretical conversion factor from DTIV output to apparent charge is 1/(RZC_1_), i.e., Q ≈ [1/(RZC_1_)]·DTIV. Equivalently, when DTIV output is regressed as the dependent variable against reference charge, the through-origin slope is RZC_1_. The experimental DTIV-versus-charge slopes are on the order of 10^−9^–10^−8^, which supports the proportional relationship while keeping the regression slope distinct from the reciprocal charge-conversion factor.

Different integration windows and window thresholds lead to different integral values and, consequently, different calibrated scaling coefficients. Therefore, this paper uses the first and second voltage zero-crossings as integration upper limits, and uses 1% and 5% of the signal peak as integration-window thresholds. Reference charge is used as the independent variable and integral output as the dependent variable in through-origin linear regression to quantitatively evaluate the goodness of fit under each integration criterion.

**Figure 2:**
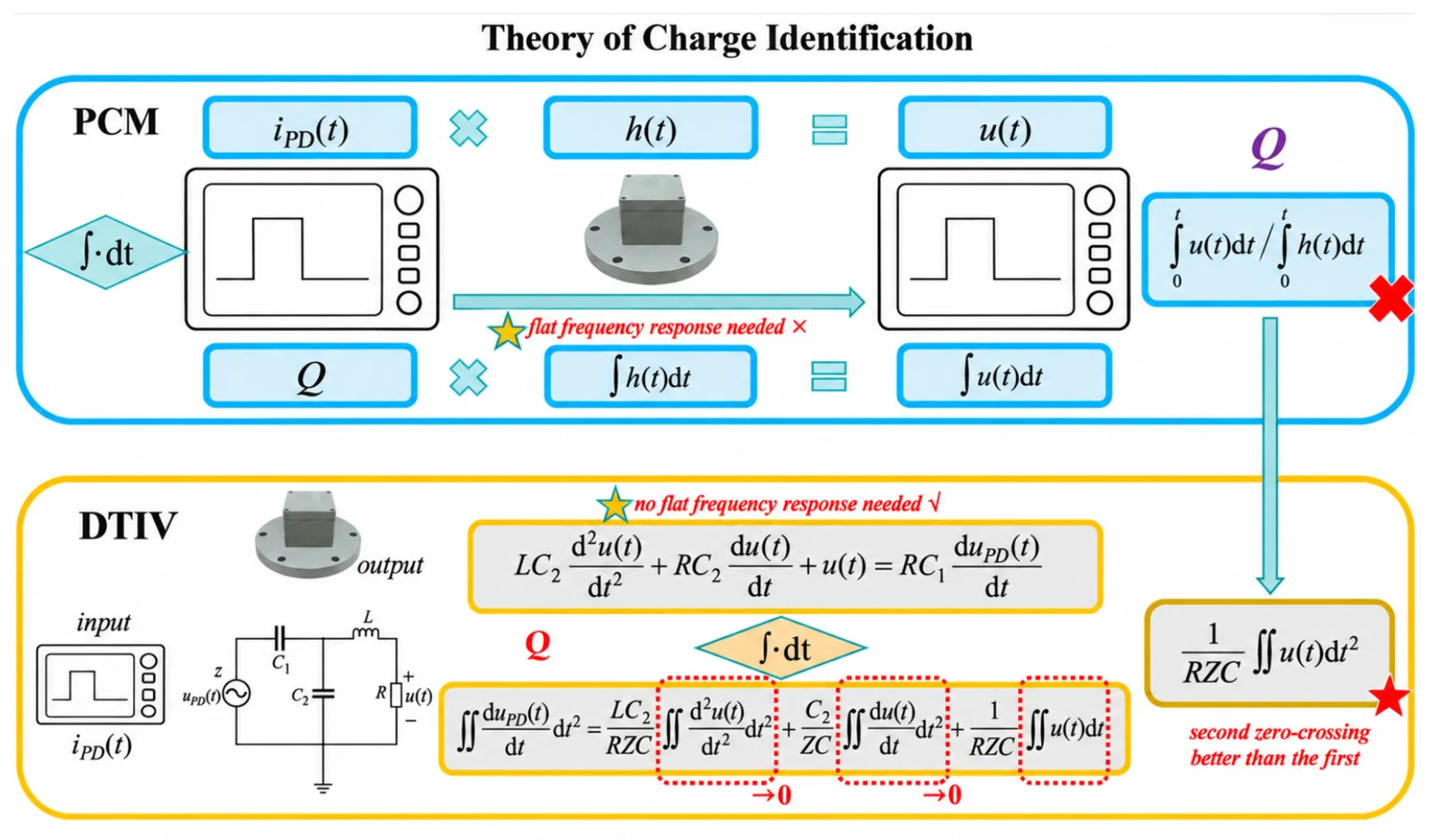
Schematic diagram of the theoretical framework. Abbreviations: PCM, pulse current method; DTIV, double time integral of voltage; PD, partial discharge.

The theoretical framework of the proposed method is shown in Figure 2.

The circuit relation also describes parameter sensitivity. For small parameter changes, the first-order gain error is

$$\frac{\Delta \hat{Q}}{Q}\approx \frac{\Delta R}{R}+\frac{\Delta Z}{Z}+\frac{\Delta C_{1}}{C_{1}}.$$

This expression describes the gain contribution only. The zero-initial-state transfer function and its natural frequency and damping ratio are

$$H(s)=\frac{U(s)}{I_{\mathrm{PD}}(s)}=\frac{\mathrm{RZ}C_{1}s}{LC_{\Sigma }s^{2}+RC_{\Sigma }s+1},$$

$$\omega_{n}=\frac{1}{\sqrt{LC_{\Sigma }}},\zeta =\frac{R}{2}\sqrt{\frac{C_{\Sigma }}{L}}.$$

Changes in *R* affect gain and damping, whereas changes in *L* or *C*_Σ_ alter the transient response and selected endpoint. *C*_1_ contributes to both the gain and *C*_Σ_. Disk diameter and installation gap influence the equivalent capacitances and field distribution. The following simulations and experiments therefore evaluate window selection for the specified sensor geometry and load.

## 3. Materials and Methods: Simulation Experiment and Data-Analysis Strategy

To achieve continuous impedance transition at the input of the GIS chamber, a conical taper with a fixed input impedance of 50 Ω is designed. The taper enables smooth transition from the input port to the GIS chamber, reduces port reflection, and provides an ideal simulation environment for charge estimation.

The structure adopts a sandwich configuration of a coaxial cone–simulated GIS–coaxial cone. The busbar and enclosure at both ends of the GIS chamber are connected by inner and outer cones, respectively. The diameter is continuously tapered from the approximately 6.2 mm N-type connector to the approximately 415 mm diameter of the simulated GIS enclosure, forming a low-reflection transition. The cone length is 500 mm and the material is aluminum. To match the 50 Ω input-port impedance, the radius ratio between the GIS enclosure and busbar satisfies:

$$Z_{0}=\frac{1}{2\pi }\sqrt{\frac{\mu }{\varepsilon }}\ln(\frac{b}{a})\Rightarrow \frac{b}{a}=e^{2\pi Z_{0}\sqrt{\varepsilon /\mu }}\approx 2.3.$$

**Figure 3:**
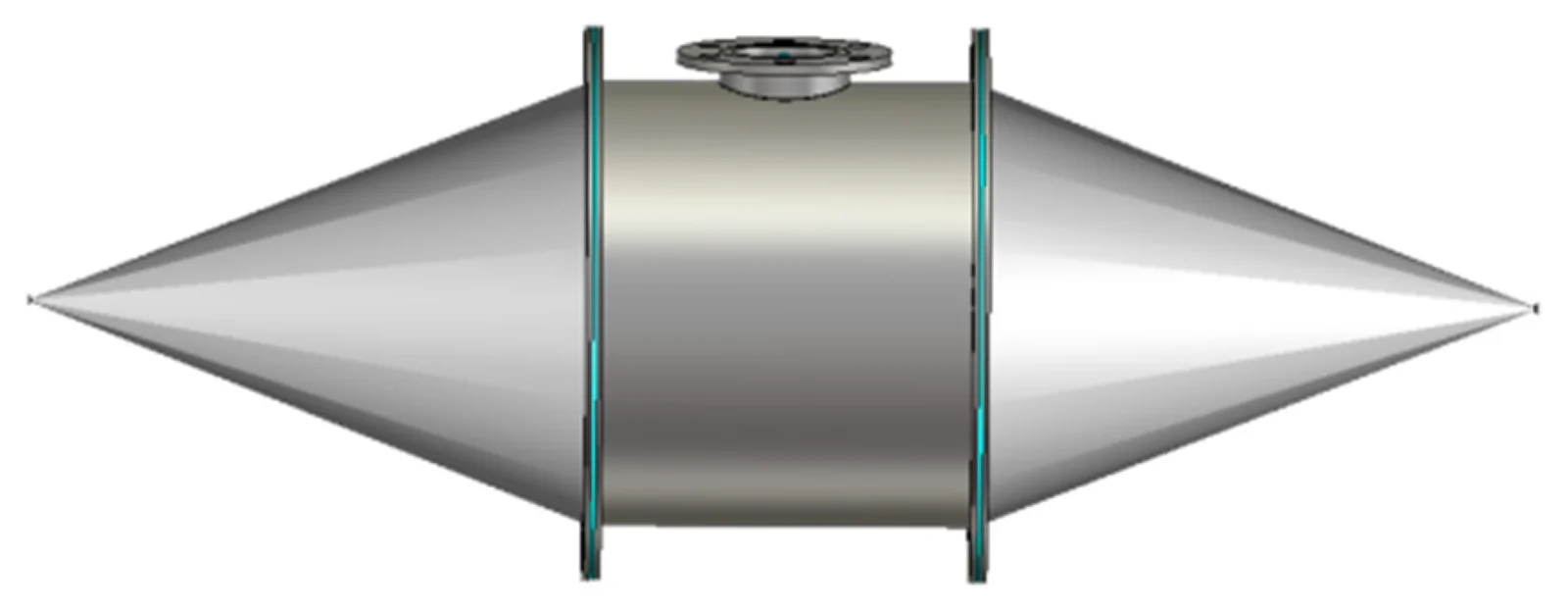
Schematic diagram of the conical-taper simulation model.

A simulation model is established in CST Studio Suite 2023, as shown in Figure 3. The GIS chamber is parametrically modeled with dimensions of 360 mm × 415 mm (GIS-section length × enclosure diameter). The material is set as a perfect electric conductor (PEC), the background material is air (ε_r_ = 1), 50 Ω waveguide ports are placed at both ends, and the frequency sweep range is 0.1–1 GHz.

The above simulation settings mainly consider TEM propagation, ideal conductors, air dielectric, and 50 Ω port matching. Practical factors such as contact resistance, connector parasitics, dielectric loss, and external electromagnetic interference are not included, so that the feasibility of the charge-estimation method can be tested in an ideal environment.

To evaluate the effect of the taper, a comparative GIS chamber model without the taper is established, and internal reflection is analyzed by comparing S-parameters. Here, S_11_ reflects the input-port reflection level, whereas S_21_ reflects the transmission capability from the input port to the output port.

**Figure 4:**
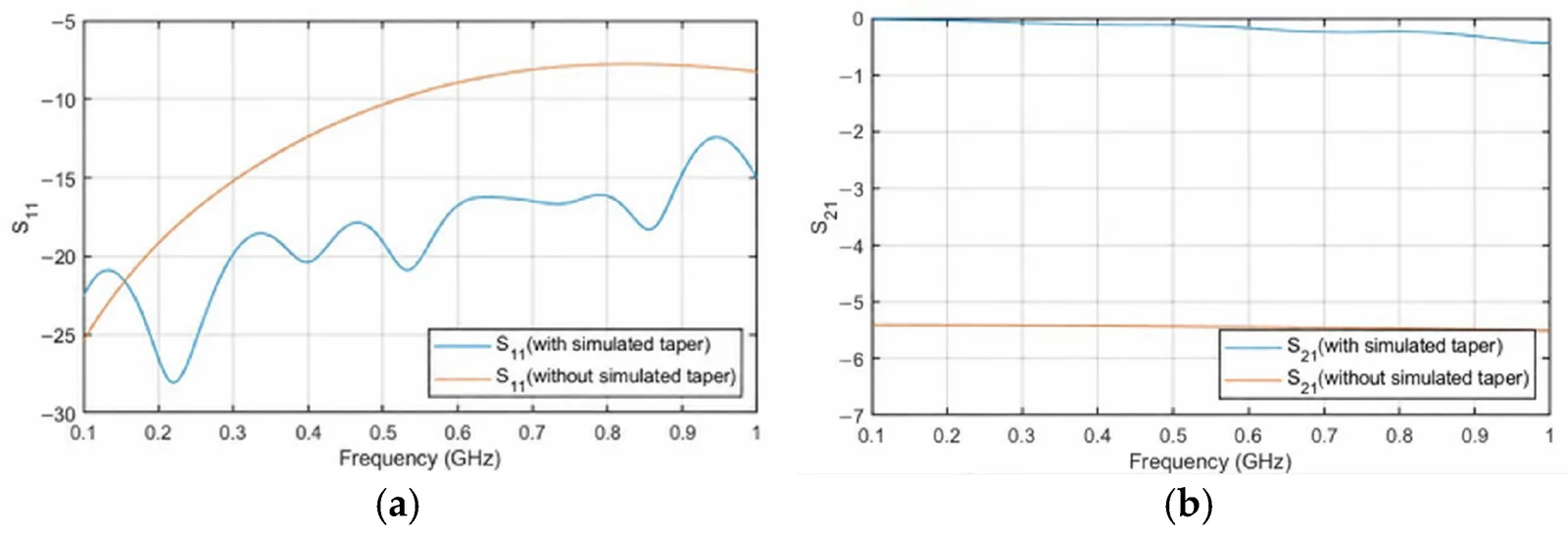
S-parameter results of the taper structure with and without the conical section: (**a**) S_11_ parameters before and after removing the conical section of the taper model; (**b**) S_21_ parameters before and after removing the conical section of the taper model.

Figure 4 shows the simulated S_11_ and S_21_ parameters before and after removing the conical part from the CST model. The results show that the S_11_ of the tapered model remains below −20 dB in the TEM-dominant region. The S_21_ fluctuation remains within approximately 1 dB, verifying that the test bench has good transmission-matching performance in the VHF band. In contrast, the S_11_ of the model without the taper increases significantly, and S_21_ exhibits an approximately 5 dB drop, indicating clearly inferior transmission performance.

**Figure 5:**
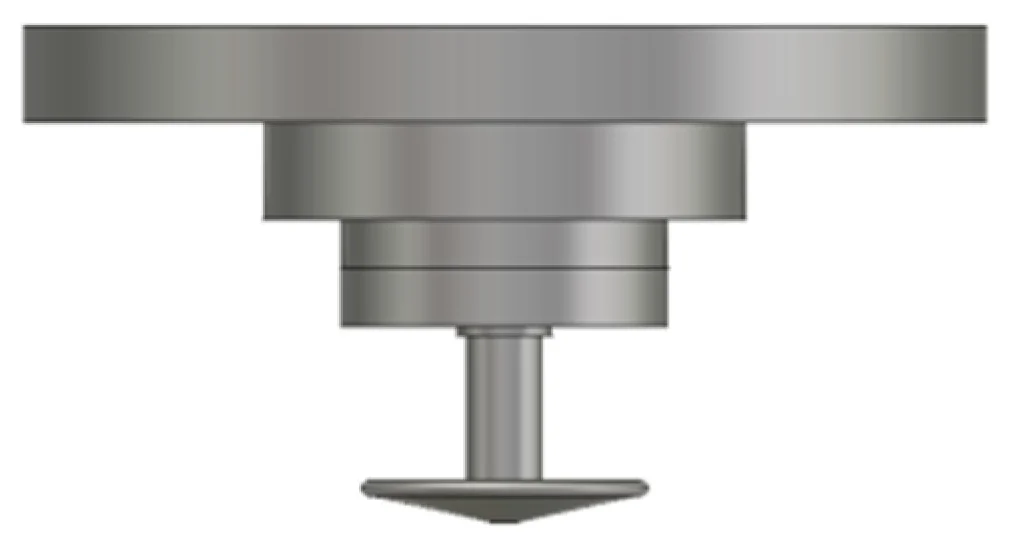
Reference model of the VHF sensor.

Figure 5 shows the VHF sensor model used in the simulation. To systematically evaluate the linear relationship between the proposed integral output and apparent charge, sensors are arranged on the validation platform and two groups of simulation experiments are designed. Reference apparent charge is taken as the horizontal axis and algorithm output as the vertical axis for linear regression, thereby characterizing the charge-estimation accuracy of the DTIV method.

## 4. Results and Discussion: Key Parameters Affecting the DTIV Method

To systematically analyze the influence of DTIV integration parameters, this paper investigates the integration window and window threshold. The integration endpoint controls finite-window truncation, while the amplitude qualification threshold controls which candidate zero crossings are accepted.

### 4.1. Integration-Window Setting of the DTIV_ZC Method

#### 4.1.1. Integration-Window Definition

The first and second accepted voltage zero crossings (ZC1 and ZC2) are compared as DTIV integration endpoints. As shown in Section 2.2, a later crossing can include an additional response lobe and reduce the remaining single integral. The benefit depends on the waveform and the response accumulated within the window.

#### 4.1.2. Experimental Design for Integration-Window Evaluation

Analytical pulses and recorded PD waveforms are imported into the CST model to examine the effects of waveform shape, amplitude, and duration. The input library contains 166 waveforms, including the following:

(1) Ten Gaussian pulses: signal peaks are 0.10 A and 0.01 A, and half-widths are 0.50 ns, 1.00 ns, 2.00 ns, 4.00 ns, and 8.00 ns. These pulses are abbreviated as “Gaussian” in the tables. The half-width at half maximum (HWHM) is the time interval from the pulse peak to the half-maximum point on either side, and equals half the full width at half maximum (FWHM).
(2) Ten rectangular pulses: signal peaks are 0.10 A and 0.01 A, the width is 10.00 ns, and rise-edge widths are 0.25 ns, 0.50 ns, 1.00 ns, 2.00 ns, and 4.00 ns. These pulses are abbreviated as “Rectangular” in the tables.
(3) Ten triangular pulses: signal peaks are 0.10 A and 0.01 A, and rise-edge widths are 0.25 ns, 0.50 ns, 1.00 ns, 2.00 ns, and 4.00 ns. These pulses are abbreviated as “Triangular” in the tables.
(4) Eighteen exponentially decaying pulse functions are generated using the following general expression:
  $$I_{\mathrm{PD}}=I_{0}(t-t_{0}{)}^{n}e^{-k(t-t_{0})/\tau }.$$

I_0_ is set to 1.2649 × 10^−2^ A, 1.2649 × 10^−3^ A, and 1.2649 × 10^−4^ A; the time constant t_0_ is set to 0; the power exponent n is set to 0.50 and 1.00; the attenuation coefficient k is set to 0.05; and the decay time constants are 0.01, 0.05, and 0.10. These pulses are abbreviated as “Exponential 1” in the tables.

(5) Eighteen sinusoidally modulated exponentially decaying pulse functions are generated using the following general expression:
  $$I_{\mathrm{PD}}=I_{0}(t-t_{0}{)}^{n}e^{-k(t-t_{0})/\tau }\sin[2\pi f_{0}(t-t_{0})]$$

I_0_ is set to 5.6437 × 10^−2^ A, 5.6437 × 10^−3^ A, and 5.6437 × 10^−4^ A; the time constant t_0_ is set to 0; the rise-edge power exponent n is set to 5 and 10; the attenuation coefficient k is set to 2; the decay time constants are 0.01, 0.02, and 0.1; and the oscillation frequency f_0_ is set to 1 GHz. These pulses are abbreviated as “Exponential 2” in the tables.

(6) One hundred typical PD pulses collected from a laboratory artificial-defect platform are imported into the CST model as an input-pulse library. The main sources include particle defects, floating-electrode defects, insulation air-gap defects, and protrusion defects; these pulses are abbreviated as “Artificial” in the tables.

MATLAB 2023 is then used to perform double time integration on each simulated output voltage. The time-integration intervals of DTIV_ZC1_ and DTIV_ZC2_ are set as [0, ZC_1_] and [0, ZC_2_], respectively. ZC_1_ and ZC_2_ are determined by a voltage zero-crossing detection algorithm.

$$Q_{\mathrm{ref}}=\int_{0}^{t}i_{\mathrm{PD}}\left(t\right)\mathrm{dt};$$

$$Q_{\mathrm{DTIV}\_\mathrm{ZC}1}=\int_{0}^{\mathrm{ZC}1}\int u(t)dt^{2};$$

$$Q_{\mathrm{DTIV}\_\mathrm{ZC}2}=\int_{0}^{\mathrm{ZC}2}\int u(t)dt^{2}.$$

The fitted slope k relates the voltage double integral S to reference charge Q_ref_ and has units of Ω·s. Q_ref_ is calculated by the basic definition of the pulse current method. R^2^ is the coefficient of determination for the stated regression convention, and ε is the mean absolute relative error.

After obtaining the two groups of data, linear regression is performed to obtain the calibrated slopes kZC_1_ and kZC_2_, coefficients of determination R^2^_ZC1_ and R^2^_ZC2_, and relative errors ε_ZC1_ and ε_ZC2_.

The 1% and 5% thresholds were used as amplitude qualification gates. After pre-trigger baseline subtraction, ZC1 was defined as the first accepted sign reversal after the dominant peak and ZC2 as the following accepted sign reversal. Linear interpolation between the two bracketing samples was used to determine the crossing time.

#### 4.1.3. Results and Discussion of Integration-Window Evaluation

**Figure 6:**
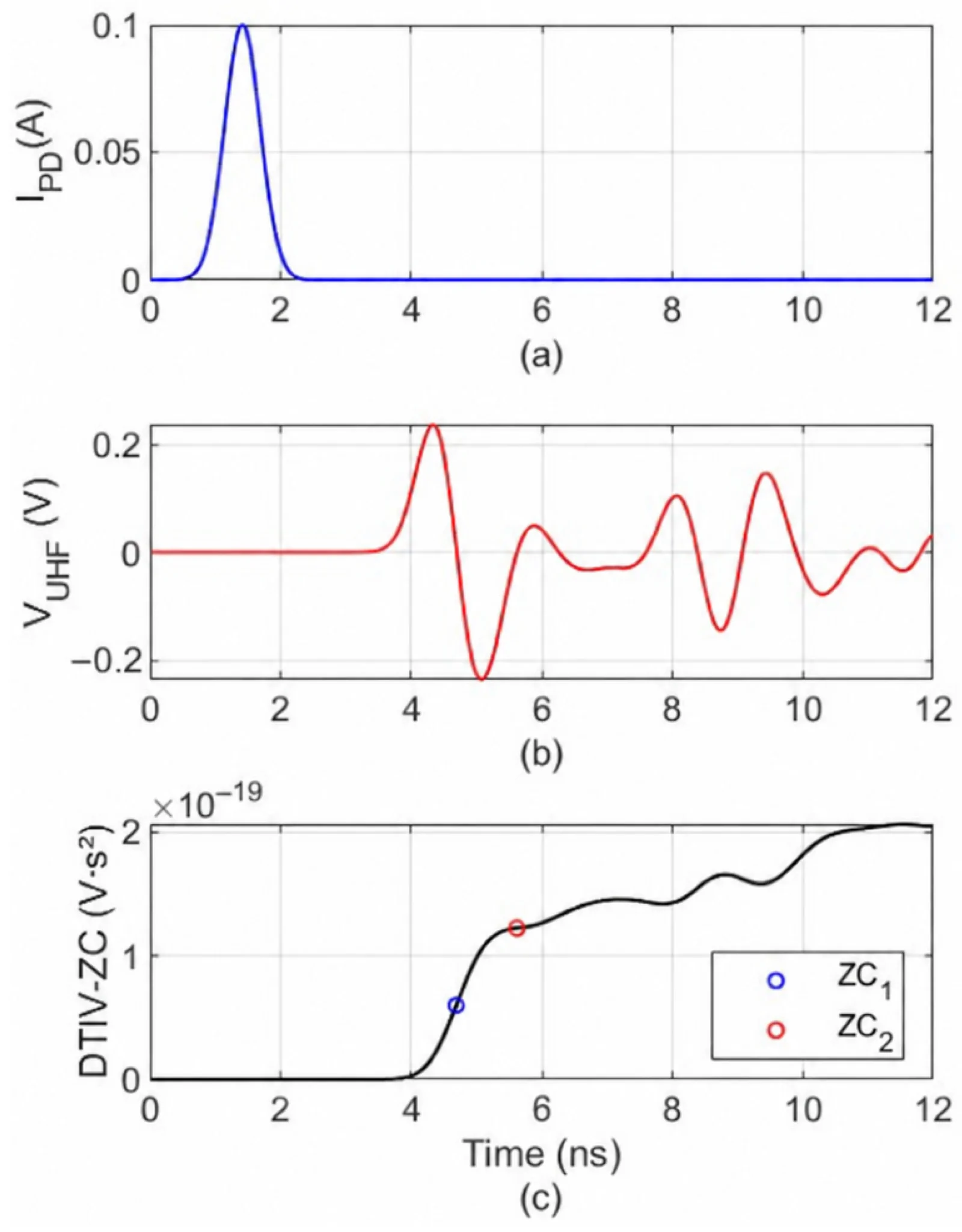
Simulated sensor output voltage and DTIV double-integration result for a Gaussian pulse: (**a**) original Gaussian pulse, (**b**) sensor output signal, and (**c**) double-integration curve obtained by the DTIV method.

**Figure 7:**
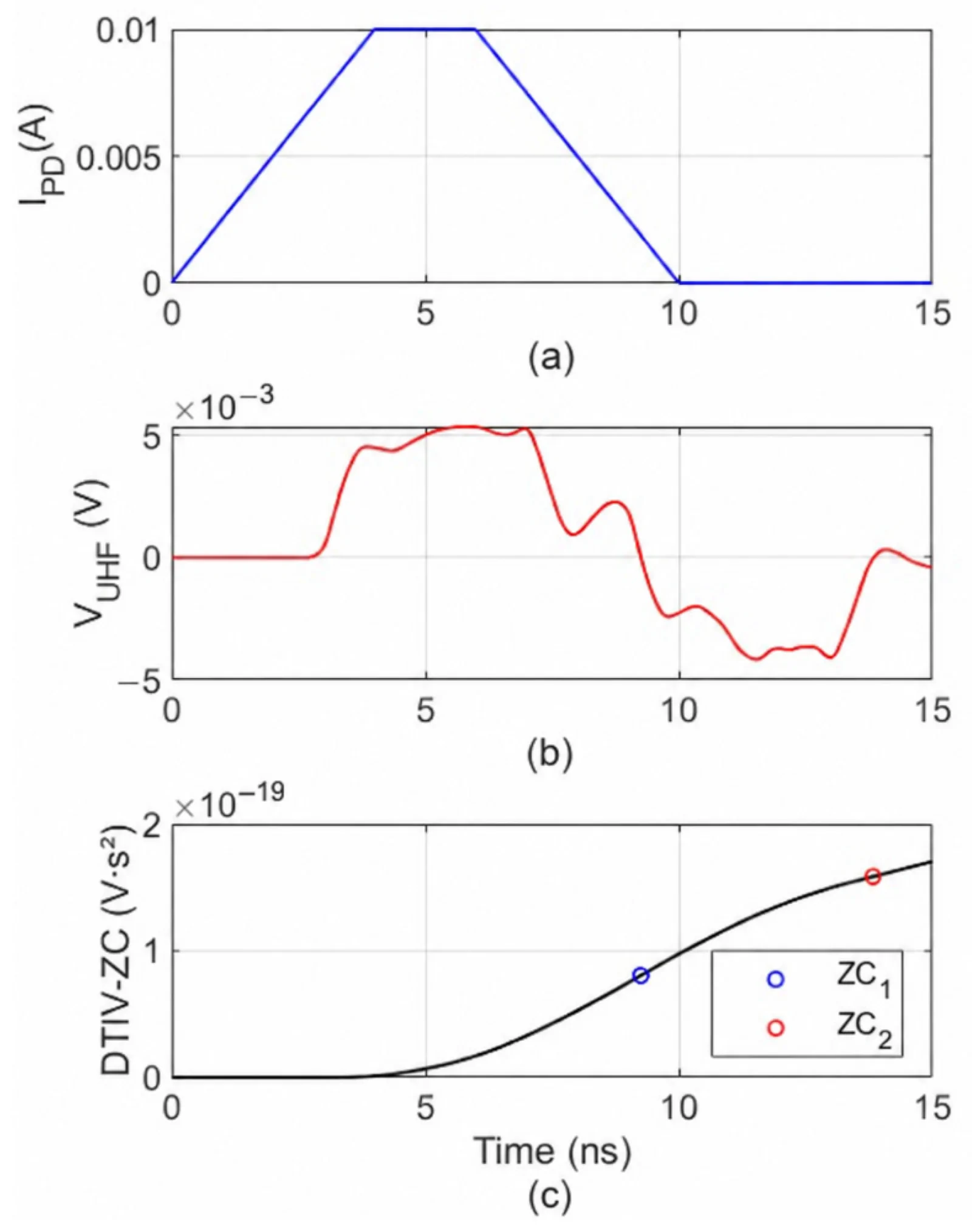
Simulated sensor output voltage and DTIV double-integration result for a rectangular pulse: (**a**) original rectangular pulse, (**b**) sensor output signal, and (**c**) double-integration curve obtained by the DTIV method.

**Figure 8:**
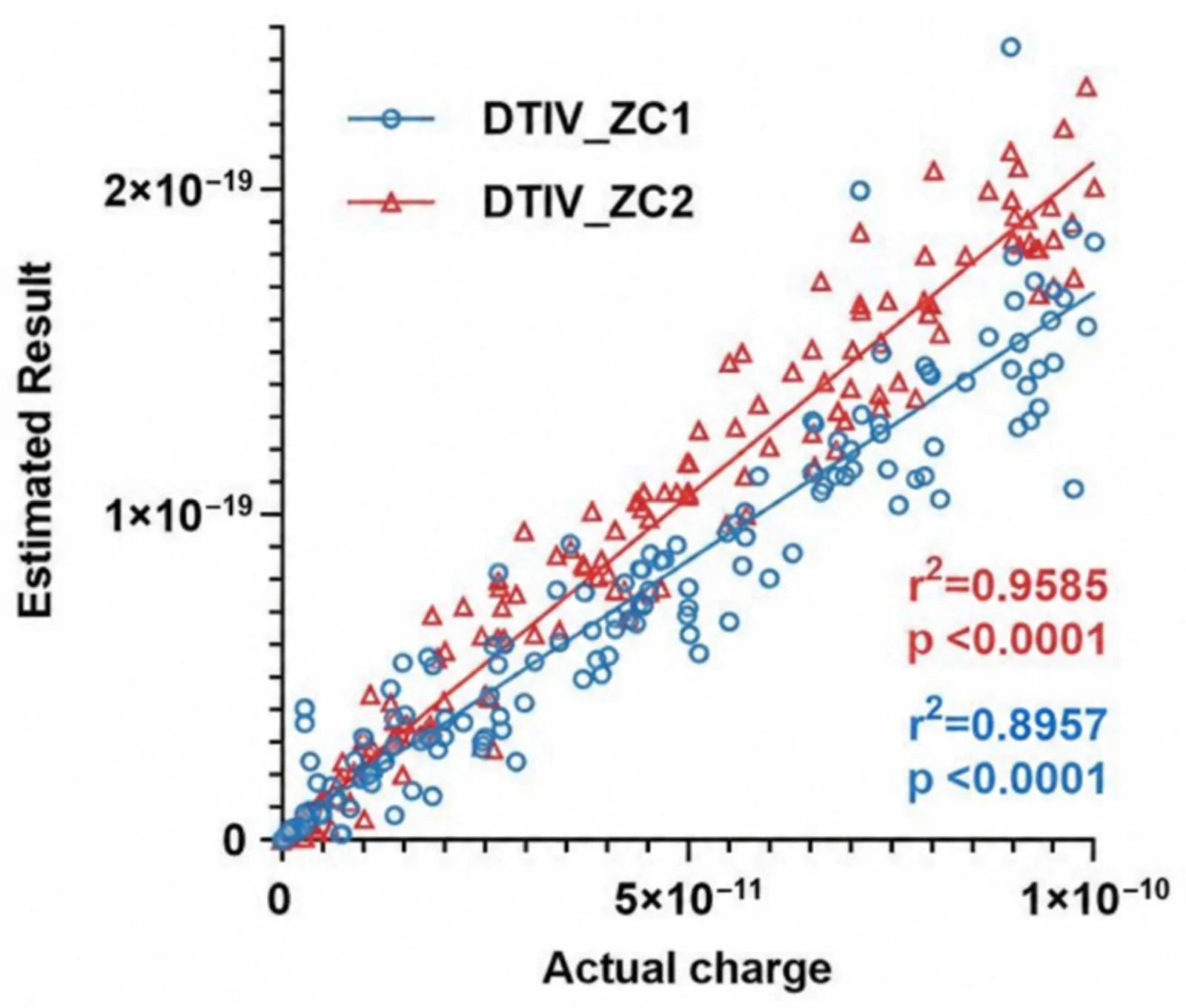
Linear-regression results between DTIV_ZC1_/DTIV_ZC2_ integral output and reference charge.

Figure 6 and Figure 7 show the output voltage and DTIV integration results for Gaussian and rectangular pulses, respectively. After calculation and statistical analysis, Figure 8 presents the scatter distributions obtained by linearly regressing the integral quantities of the DTIV_ZC1_ and DTIV_ZC2_ methods against the reference charge.

Figure 8 shows the scatter distributions of integral output versus reference charge under the two integration truncation strategies, and Table 1 summarizes the corresponding indicators. For DTIV_ZC1_, the fitted slope for Gaussian pulses is 1.78 × 10^−9^. Across all 166 samples, the overall coefficient of determination is 0.8957 and the average relative error is 7.6%. The rectangular group has a coefficient of determination of 0.8124 and an average relative error of 11.2% for ZC_1_, indicating a stronger waveform dependence than the aggregate result alone suggests. By contrast, the fitted slopes of DTIV_ZC2_ for different pulse types remain stable on the order of 2 × 10^−9^; across all samples, the overall coefficient of determination increases to 0.9585 and the average relative error decreases to 4.5%.

Considering the rectangular-pulse behavior, it can be interpreted using the finite-window relation in Section 2.2. If the source pulse is not complete, the omitted source charge adds a further error. The leading and trailing edges of a rectangular pulse excite time-shifted responses with opposite signs. Their overlap depends on the pulse duration and sensor dynamics, so a voltage zero crossing can occur while a non-negligible single-integral residual remains.

In addition, abrupt edges can be sensitive to the finite time step, mesh resolution, and output sampling interval, potentially introducing edge distortion and integration error.

Table 2 reports the relative-error distribution of charge estimates under the two truncation strategies. For DTIV_ZC1_, approximately 78.92% of samples have errors below 10%, whereas 9.03% exceed 25%. In contrast, DTIV_ZC2_ increases the proportion of samples with errors below 10% to 87.95% and reduces the proportion above 25% to 2.41%. Overall, DTIV_ZC2_ outperforms DTIV_ZC1_, and ZC_2_ is therefore adopted as the DTIV integration upper limit in the following sections.

### 4.2. Zero-Crossing Window-Threshold Setting of the DTIV_ZC Method

Section 4.1 focuses on charge-estimation performance when the first and second zero-crossings are used as DTIV integration upper limits. The comparison shows that DTIV_ZC2_ is preferable. However, abnormal regression behavior remains for signals such as rectangular pulses, mainly because of their long tails and relatively high low-frequency content. To further optimize the integration window, this section introduces different zero-crossing thresholds to suppress low-frequency tail noise while retaining the main pulse energy.

#### 4.2.1. Window-Threshold Definition

The 1% and 5% values are amplitude qualification thresholds for accepting voltage sign reversals. The integration endpoint is the interpolated time of the selected accepted zero crossing. A higher threshold makes acceptance more restrictive, and its effect on charge estimation is evaluated using the same waveform library.

#### 4.2.2. Experimental Design for Window-Threshold Evaluation

Using the CST Studio Suite platform, typical Gaussian, rectangular, triangular, and exponentially decaying input pulses are imported into the VHF sensor model. During data processing, 1% and 5% are set as zero-crossing thresholds, and the double-integral values are regressed through the origin against reference charge to evaluate charge-estimation performance under different thresholds.

#### 4.2.3. Results and Discussion for Window-Threshold Evaluation

**Figure 9:**
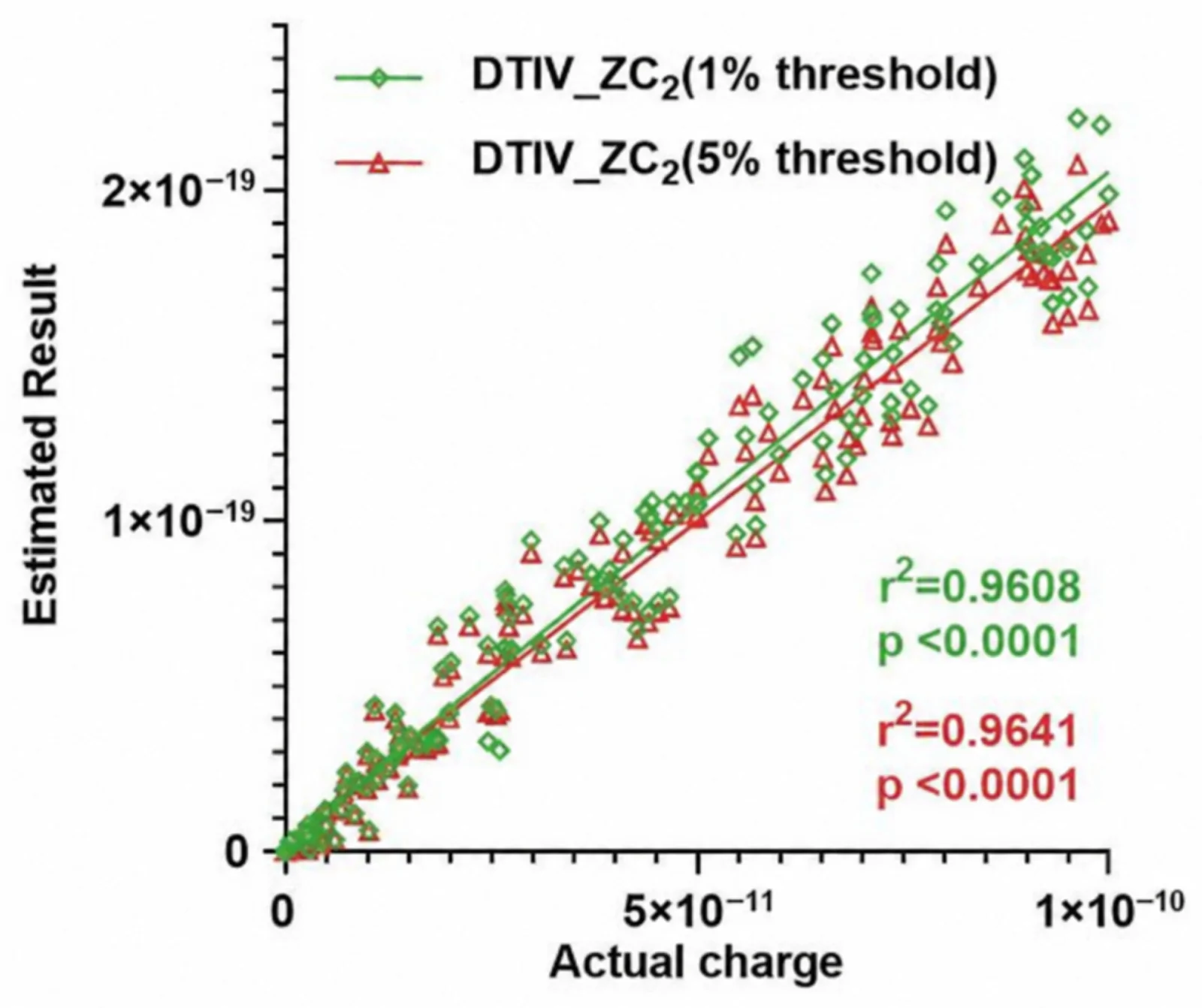
Linear-regression results of DTIV_ZC2_ under 1% and 5% peak-threshold conditions.

Figure 9 shows the linear-regression results between integral output and reference charge under different zero-crossing threshold strategies, and Table 3 summarizes the corresponding indicators. When the threshold is 1% of the peak, the overall coefficient of determination of DTIV_ZC2_ increases to 0.9608 and the average relative error decreases to 4.2%; the improvement is most evident for rectangular pulses, whose average relative error decreases to 7.2%. When the threshold is 5% of the peak, the coefficient of determination for rectangular pulses increases to 0.9032 and the average relative error decreases to 5.7%. The overall coefficient of determination increases to 0.9641 and the average relative error decreases to 3.9%.

The 5% threshold gives a lower mean error in the present library, although its effect varies with waveform. The Gaussian and Exponential 1 groups each show a 0.1 percentage-point increase in mean relative error and a small reduction in R^2^. On the basis of the results, 5% is selected for the subsequent experiments.

Table 4 reports the relative-error distribution of charge-estimation results under different zero-crossing thresholds. With the low-threshold strategy, approximately 87.95% of samples have errors below 10%, whereas 3.61% exceed 25%. In contrast, the high-threshold strategy increases the proportion of samples with errors below 10% to 90.36% and eliminates samples with errors greater than 25%. This error distribution also favors the 5% setting for the evaluated library.

Therefore, a 5% peak zero-crossing threshold is adopted as the threshold strategy for the DTIV method in this paper.

This study focuses on validating the proposed theoretical relationship under controlled conditions approximating an ideal propagation environment. The conical-transition platform reduces impedance mismatch and reflections for this purpose; a systematic assessment of additive noise and baseline drift is outside the primary scope of the present study.

To describe how these disturbances can affect the estimate, consider an additive perturbation n(t). At a fixed endpoint T, its contribution to the double integral is

$$\Delta S_{n}(T)=\int_{0}^{T}(T-t)n(t)\mathrm{dt}.$$

For a residual constant baseline b_0_ and a linear drift b_1_t, this gives

$$n(t)=b_{0}+b_{1}t⟹\Delta S_{n}(T)=\frac{b_{0}T^{2}}{2}+\frac{b_{1}T^{3}}{6}.$$

Longer windows can therefore accumulate more baseline error. For an isolated simple zero crossing *T_c_* with nonzero slope, a small perturbation that preserves the selected crossing gives

$$\delta T_{c}\approx -\frac{n(T_{c})}{u^{\prime }(T_{c})},\Delta S_{\mathrm{endpoint}}\approx J(T_{c})\delta T_{c}.$$

The endpoint contribution follows from *S*^′^(*T*) = *J*(*T*). Together, these expressions describe baseline accumulation and the timing sensitivity of a selected crossing. Larger disturbances may change which crossing is accepted. Pre-trigger baseline subtraction and crossing-time interpolation address baseline offset and sampling resolution, respectively; residual drift and interference require additional treatment.

### 4.3. Summary of Simulation Results

This section constructs an input-pulse library consisting of Gaussian, rectangular, triangular, sinusoidally modulated, and laboratory artificial-defect pulses, and evaluates the method by linear-regression analysis between reference charge and algorithmic integral output. The simulation results show that DTIV_ZC2_ with a 5% peak threshold achieves the highest coefficient of determination and the best linearity for the present input-pulse library and sensor model. This strategy is therefore used in the subsequent experimental validation.

## 5. Experimental Validation

The simulation results support the use of the DTIV strategy with ZC_2_ as the integration upper limit and 5% of the signal peak as the threshold criterion. To further validate its applicability in a practical measurement chain, this section establishes an experimental platform consisting of a taper, a GIS coaxial chamber, and a VHF sensor, and designs two types of experiments. First, controllable waveforms consistent with the simulation settings are generated by a signal generator to verify the linear relationship between integral value and equivalent charge. Second, artificial-defect signals are combined with HFCT measurements to evaluate the method under more realistic PD-like conditions.

### 5.1. Experimental Platform Configuration

**Figure 10:**
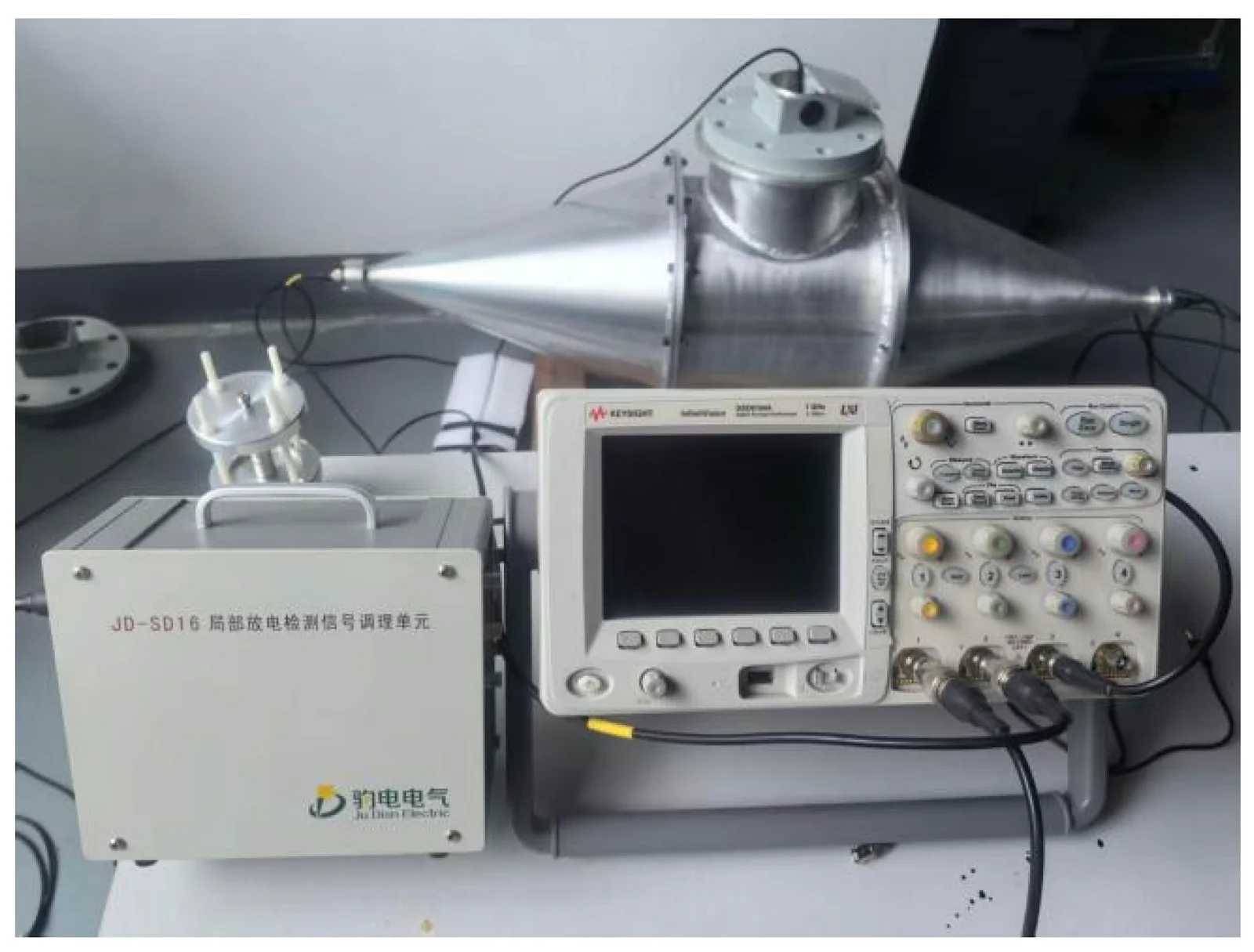
Photograph of the experimental platform.

The experimental platform consists of a signal source, tapping unit, taper, GIS coaxial chamber, VHF sensor, HFCT reference branch, signal-conditioning unit, and oscilloscope. A photograph of the experimental platform is shown in Figure 10.

The geometry, installation configuration, and measurement-chain parameters of the VHF sensor affect the sensor transfer function and hence the integration result. The sensor parameters used in this section are listed in Table 5.

**Figure 11:**
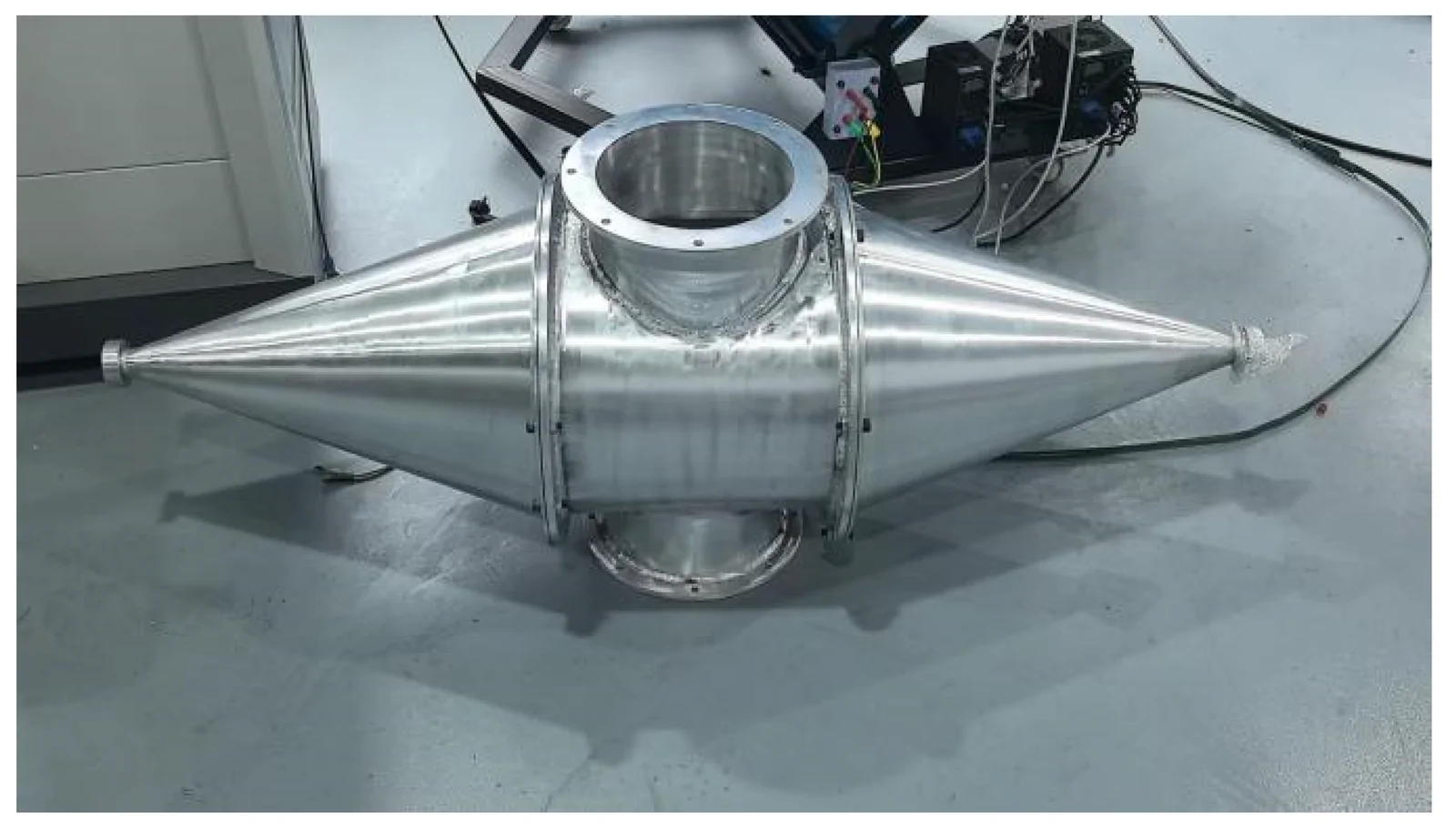
GIS simulation experimental platform.

The geometric dimensions of the GIS simulation experimental platform are consistent with the preceding simulation model, as shown in Figure 11. The platform consists of conical tapers at both ends and a central GIS section. The outer cone is fabricated from 1 mm thick rolled aluminum sheet metal, the inner cone is made of aluminum-foil-covered paperboard, the GIS section is 360 mm long, and the insulating medium inside the chamber is air.

### 5.2. Controlled-Waveform Injection Experiment

**Figure 12:**
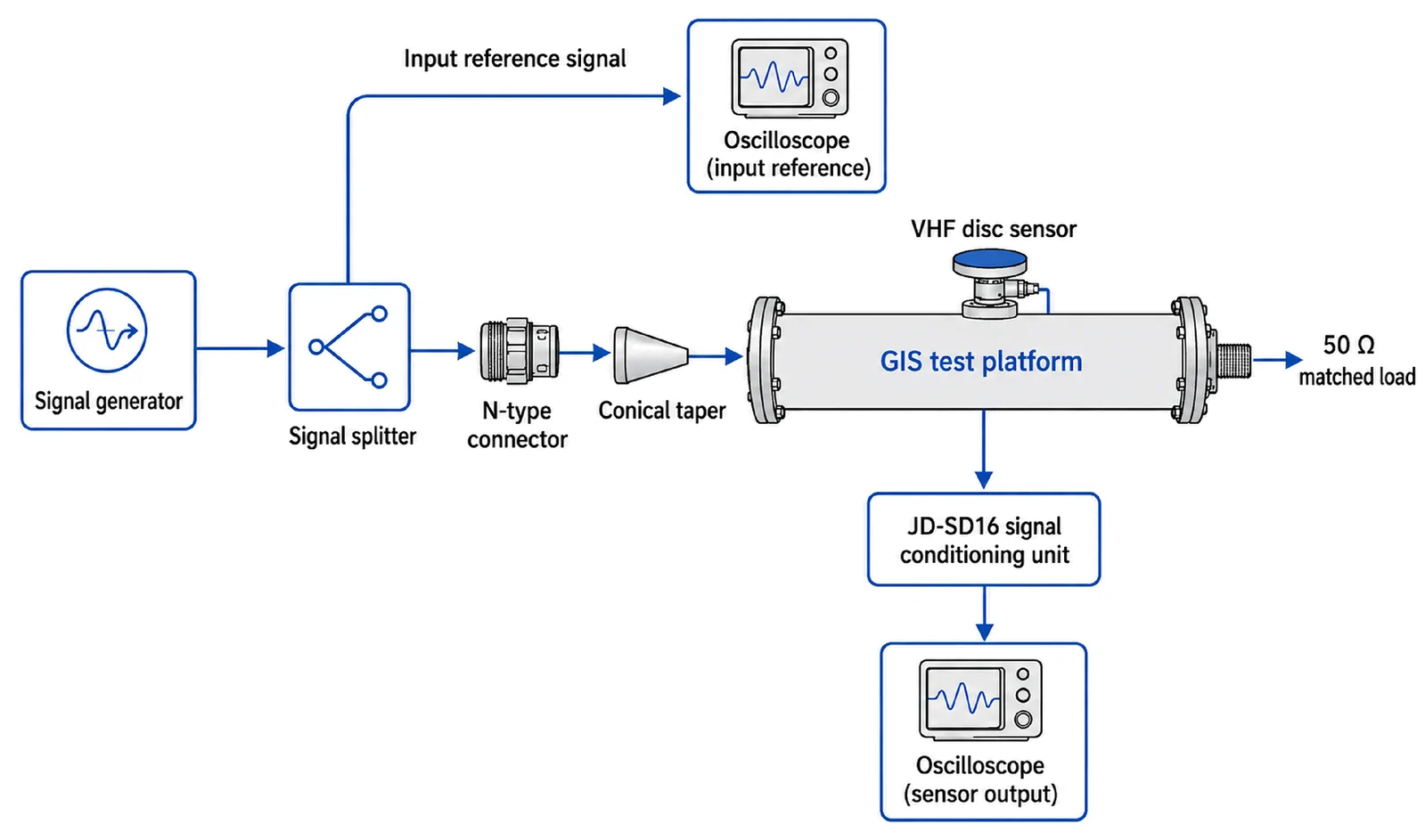
Schematic of the controlled-waveform injection test platform.

This section uses a signal generator to generate controllable input signals and verifies the mapping relationship between sensor output and input charge. One output of the signal generator is connected to the oscilloscope as the input reference, while the other is injected into the GIS experimental platform through an N-type connector and taper. A 50 Ω matched load is connected at the end of the platform, as shown in Figure 12.

**Figure 13:**
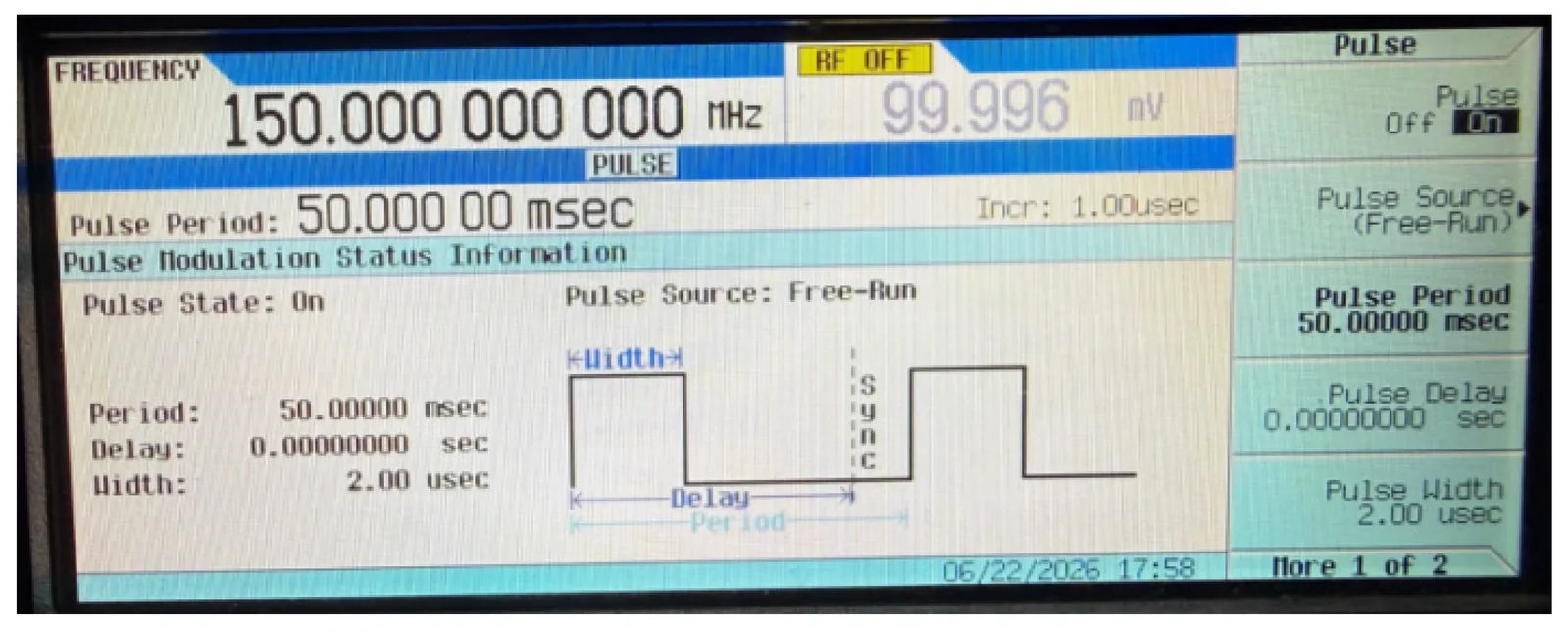
Example of signal-output parameter settings.

Gaussian, rectangular, and triangular waveforms are selected as input signals. Different equivalent input charges are obtained by adjusting the output amplitude and pulse width. Figure 13 shows an example of the signal-generator settings for a 150 MHz rectangular signal.

**Figure 14:**
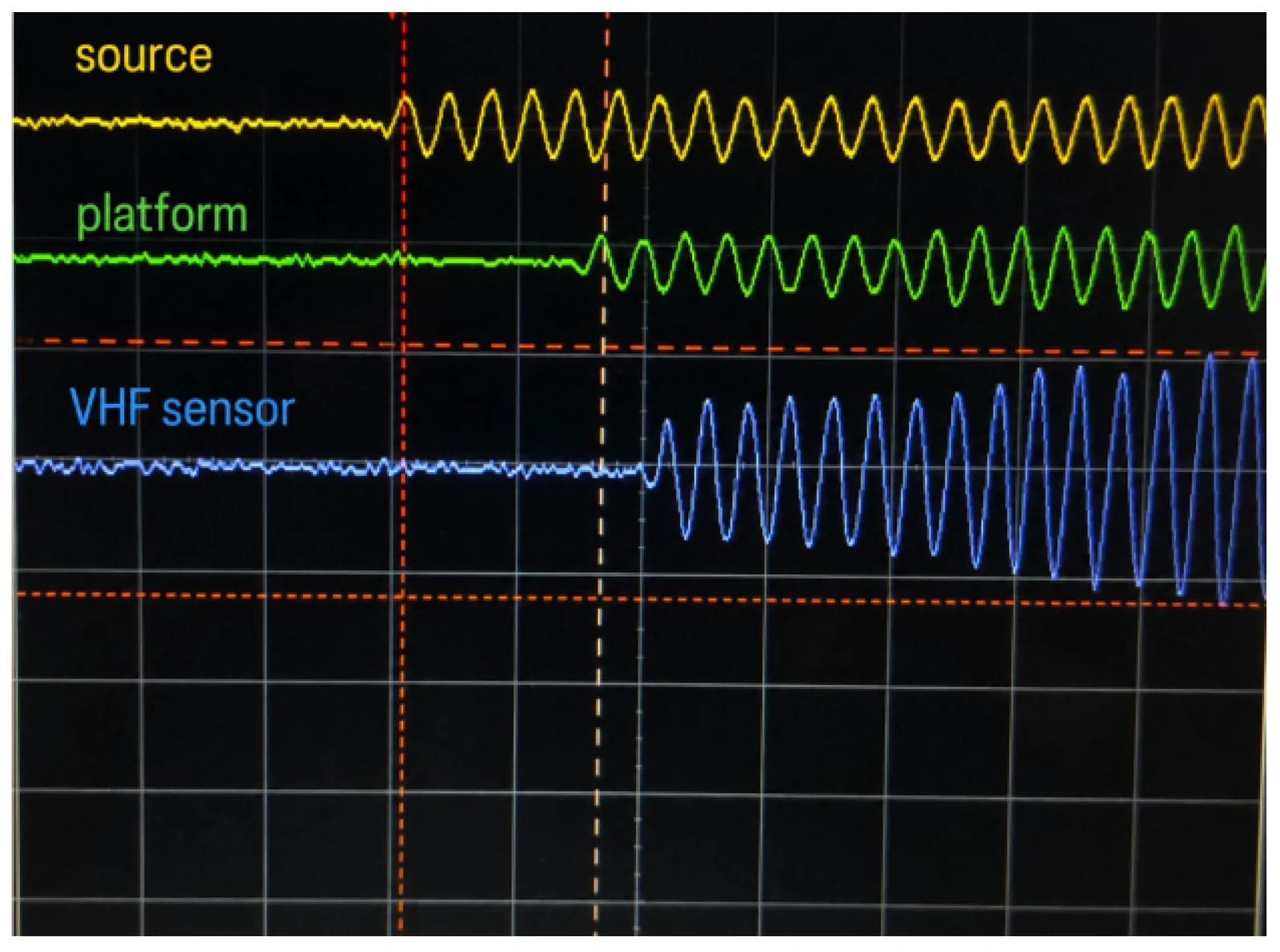
Waveforms of the signal-generator output, experimental-platform output, and VHF sensor signal.

In Figure 14, channel 1, channel 2, and channel 3 record the signal-generator output, the experimental-platform output, and the VHF sensor output after the signal-conditioning unit, respectively. The signal-conditioning unit mainly provides broadband amplification, with a nominal operating bandwidth of 300 MHz to 1.5 GHz and an approximate voltage gain of 40 dB.

The output voltage is processed by the DTIV method using ZC_2_ as the integration upper limit and 5% of the peak as the threshold criterion. A high-signal-to-noise reference pulse is selected as the calibration sample. The reference charge Q_ref1_ is calculated by PCM, and the charge-conversion coefficient k_1_ is determined from the integral value as follows:

$$k_{1}=\frac{Q_{\mathrm{ref}1}}{\int_{0}^{t_{\mathrm{ZC}2}}\left[\int_{0}^{t}u(\tau )\mathrm{d\tau}\right]\mathrm{dt}}.$$

The coefficient is then applied to the remaining integral values to obtain the estimated charges, as expressed below:

*Q_i_*_1_ = *k*_1_*S_i_*_1_.

**Figure 15:**
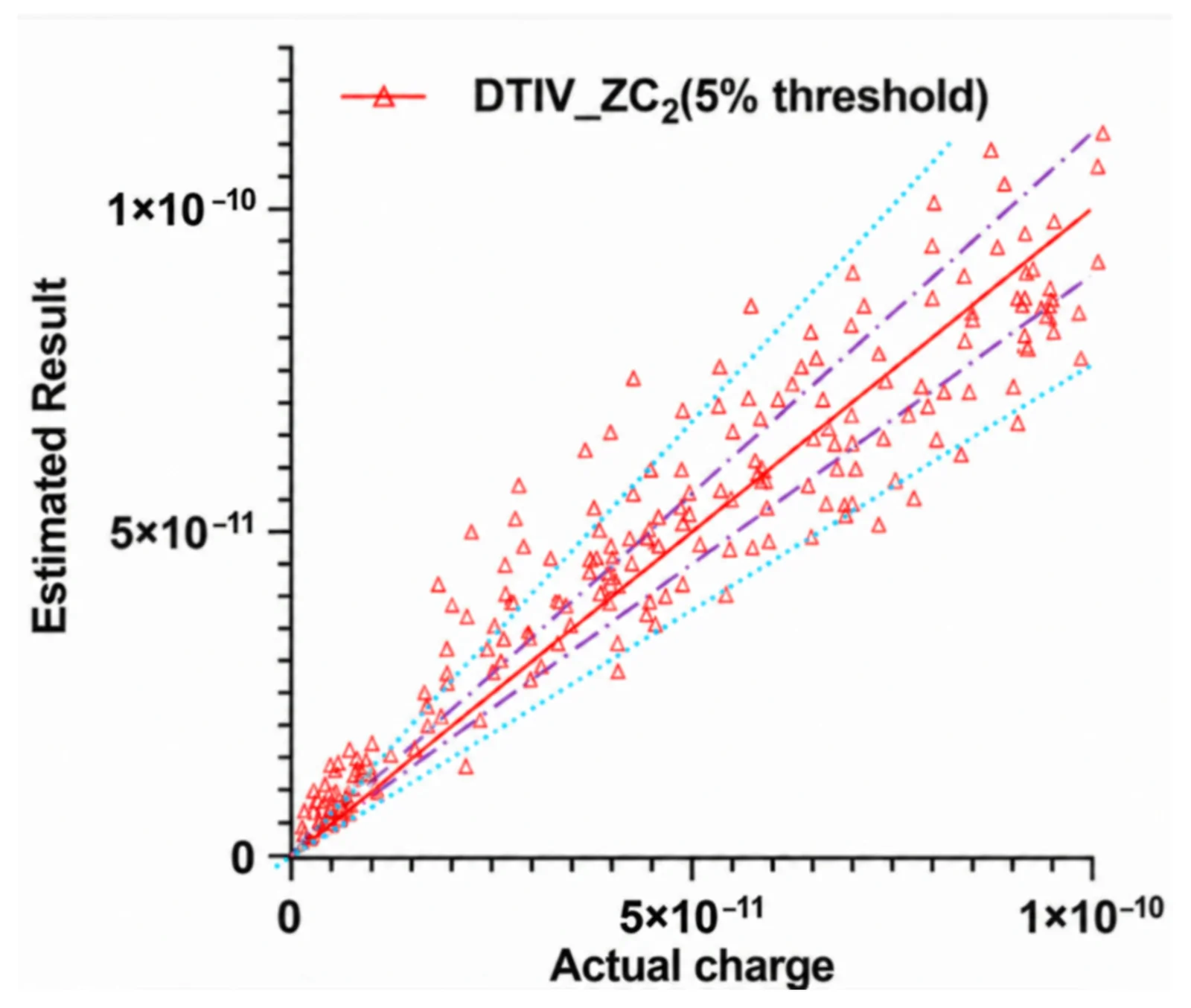
Comparison between estimated charge and equivalent input charge under controllable-waveform injection.

Finally, the estimated charges are compared with the corresponding reference charges Q_ref1_ to evaluate the error level of the charge-estimation method. Figure 15 presents the charge-estimation results for the controllable-waveform injection experiment. The purple dashed lines indicate the ±10% relative-error boundaries, while the light blue dashed lines indicate the ±25% relative-error boundaries.

The experimental results show that most samples have relative errors within 25% (blue dashed line), and many have errors below 10% (purple dash-dotted line). These results support the calibrated relationship between the VHF voltage double integral and reference charge on the laboratory platform.

### 5.3. Verification Using Artificial Defect Signals

**Figure 16:**
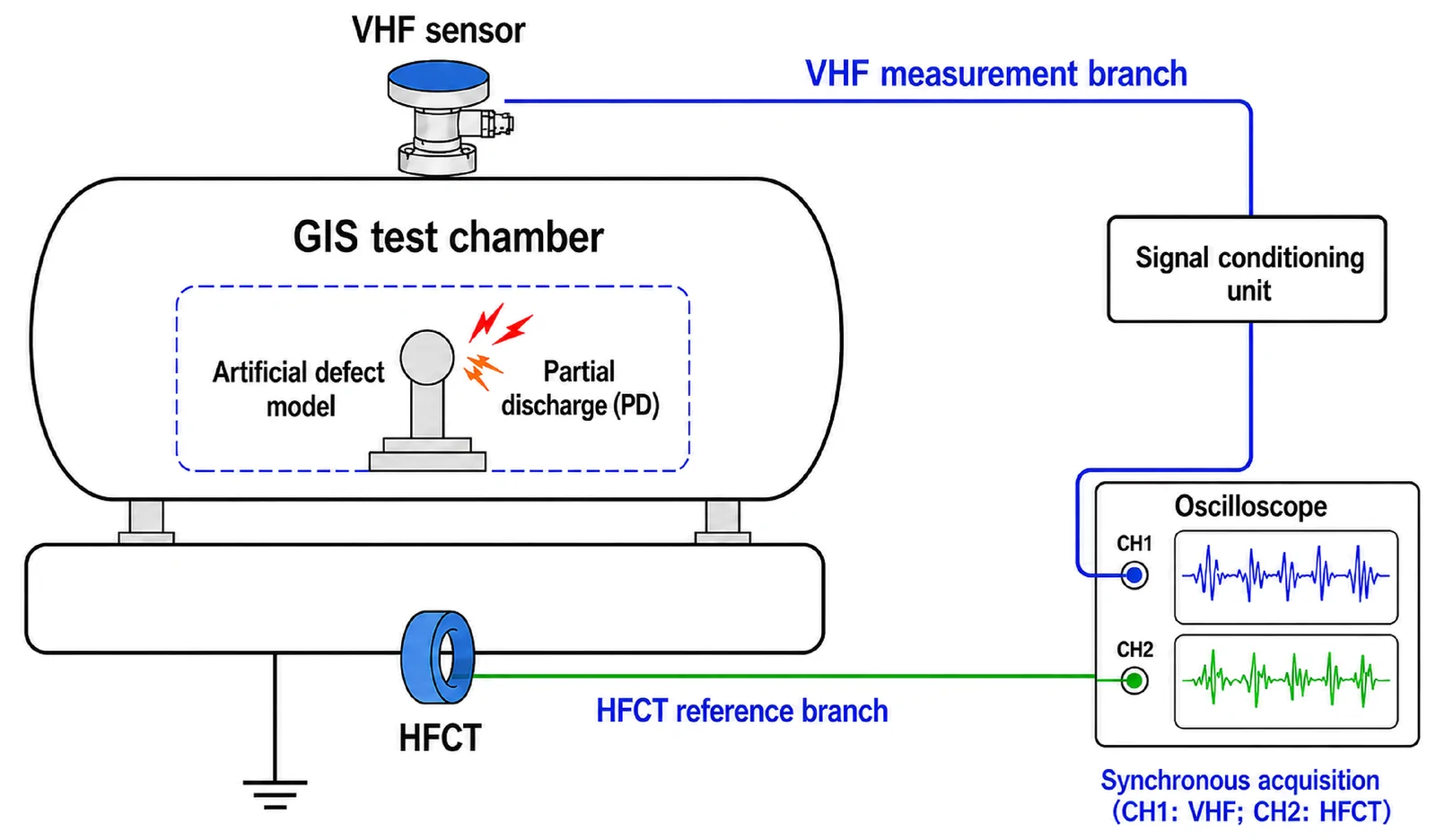
Schematic of the artificial-defect partial-discharge experiment.

The reference PD quantity in the controllable-waveform experiment is clear and the waveform is controllable. Actual PD signals, however, are stochastic. To further demonstrate the robustness of the proposed method for complex PD, artificial-defect models are used to generate artificial PD defect signals with different waveform characteristics, as shown in Figure 16.

In this experiment, the defect model generates PD inside the GIS chamber. The VHF sensor is connected to the oscilloscope through the signal-conditioning unit, while the HFCT is clamped on the grounding loop and acquired synchronously as the reference measurement branch.

**Figure 17:**
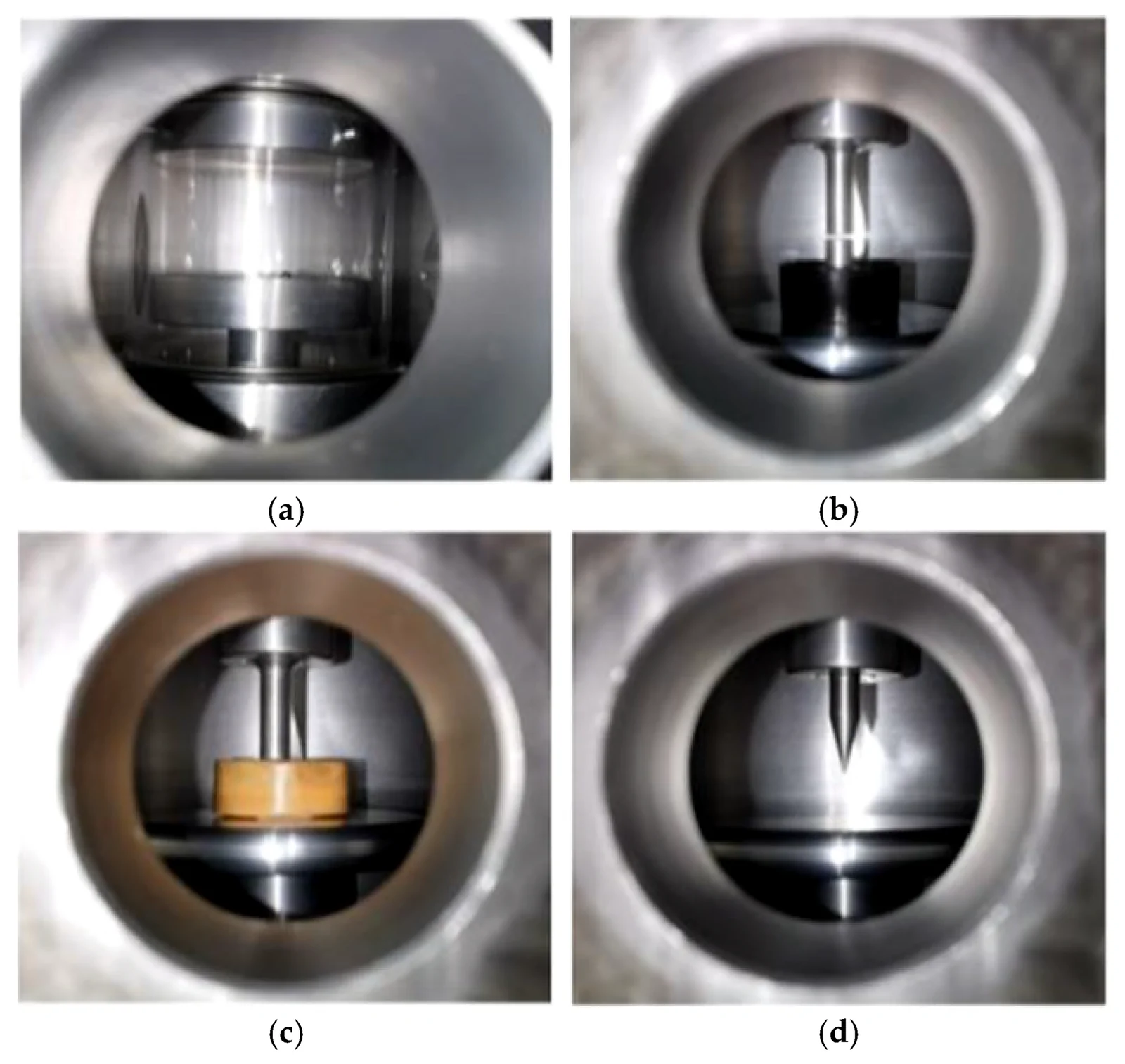
Typical artificial PD defect models and the VHF sensor: (**a**) particle discharge, (**b**) floating-electrode discharge, (**c**) insulation air-gap discharge, and (**d**) protrusion discharge.

The four defect types include particle defects, floating-electrode defects, insulation defects, and protrusion defects, as shown in Figure 17.

**Figure 18:**
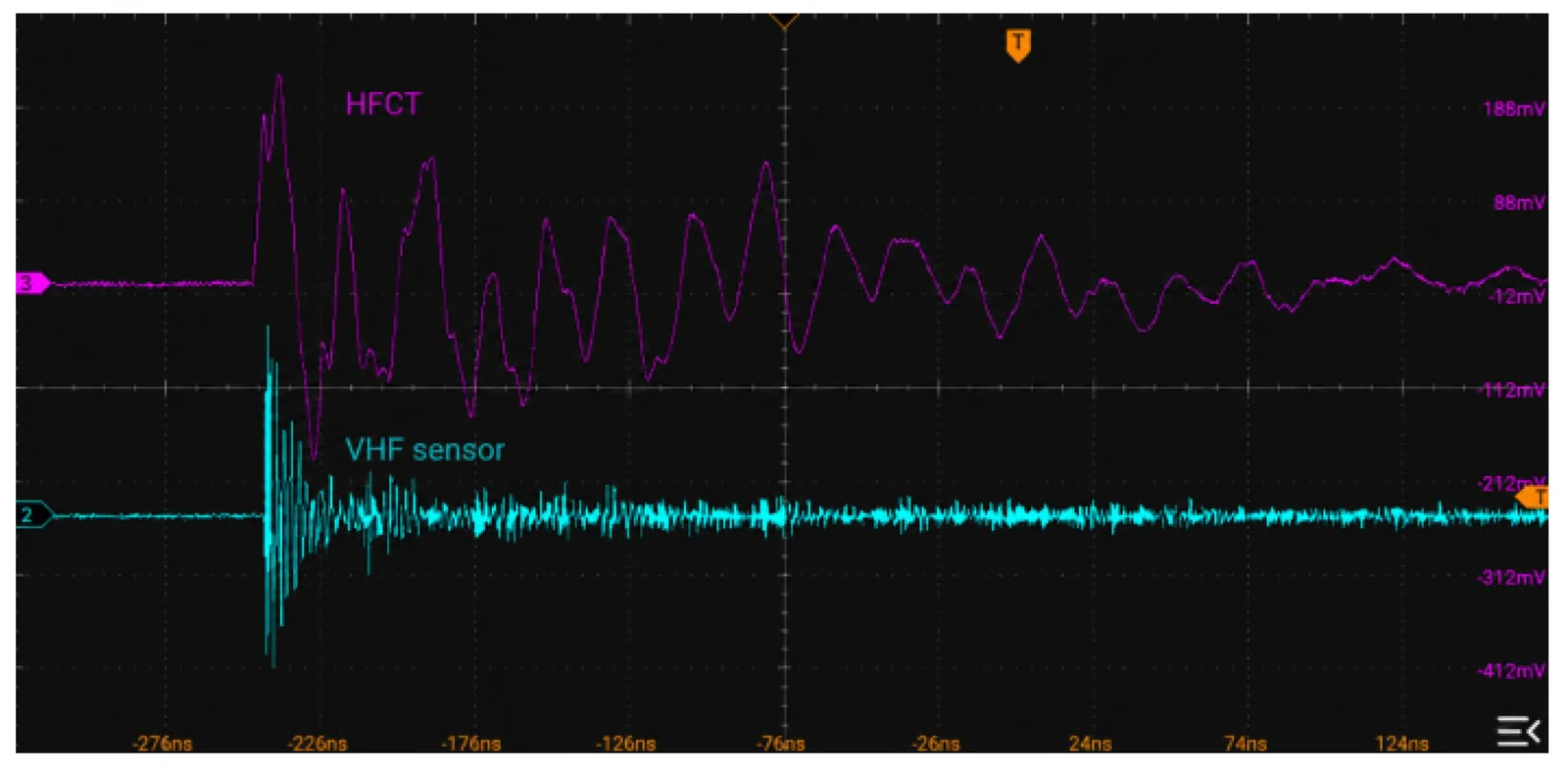
Time-domain waveforms of the HFCT and VHF sensor under artificial-defect signals.

In the experiment, the HFCT and VHF sensor acquire signals synchronously, as shown in Figure 18. The reference charge is compared with the integration result. The HFCT obtains the calibration coefficient from a selected calibration charge, thereby obtaining the reference charge Q_ref2_.

$$k_{2}=\frac{Q_{\mathrm{ref}2}}{\int_{0}^{t_{\mathrm{ZC}2}}\left[\int_{0}^{t}u(\tau )\mathrm{d\tau}\right]\mathrm{dt}}.$$

The estimated charge of the remaining samples is:

*Q_i_*_2_ = *k*_2_*S_i_*_2_.

**Figure 19:**
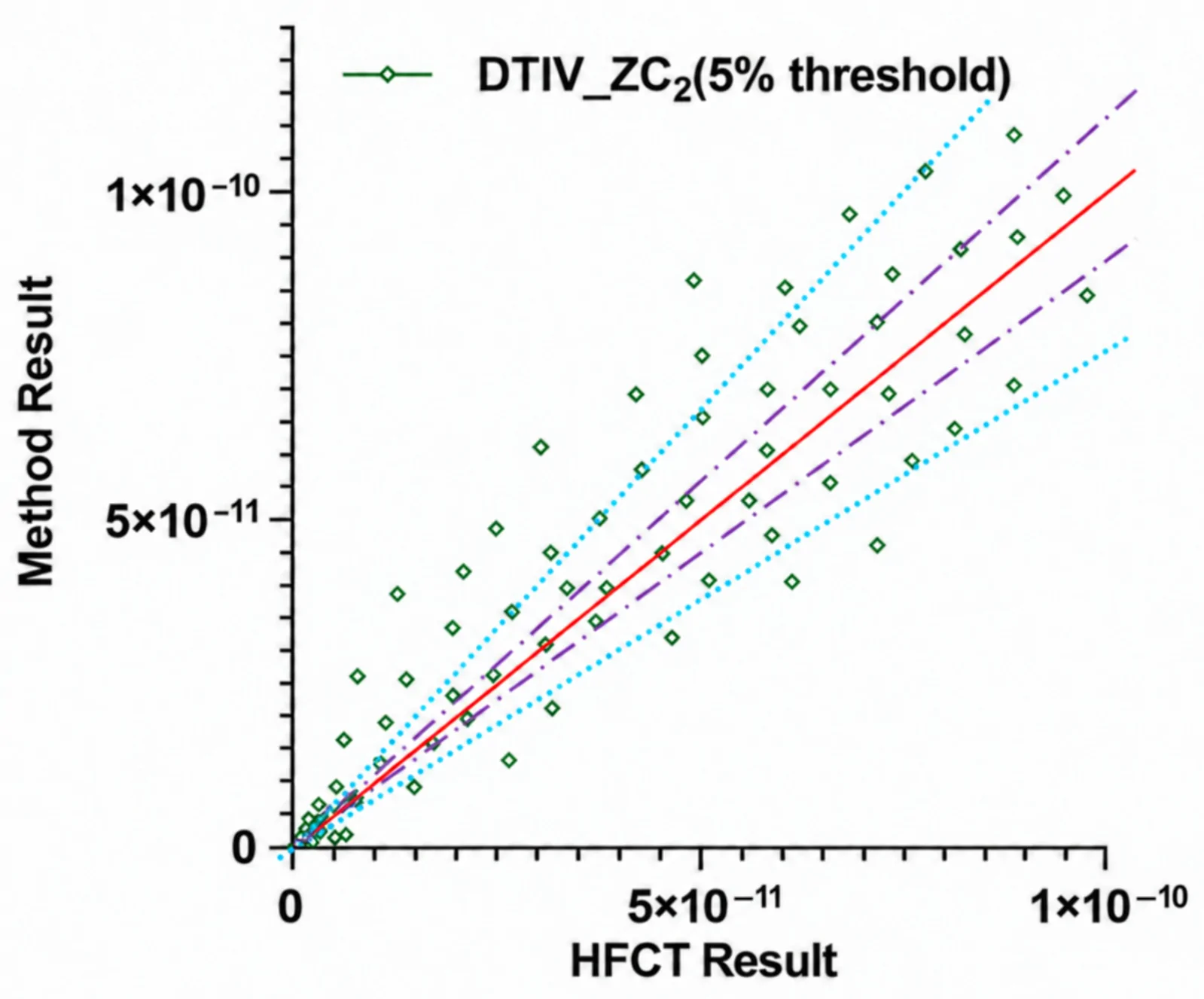
Comparison between estimated charge and HFCT reference charge under artificial-defect signals.

The estimated charge Q_i2_ is compared with the reference charge Q_ref2_. Figure 18 shows the typical waveforms of each defect signal, and Figure 19 presents the charge-estimation results for the artificial-defect signal experiment.The purple dashed lines indicate the ±10% relative-error boundaries, while the light blue dashed lines indicate the ±25% relative-error boundaries.

### 5.4. Limitations and Contributions

The results support DTIV charge estimation for the tested sensor and measurement chain. The conical transitions reduce reflections at the injection interface, while the physical artificial defect tests examine a range of discharge waveforms.

For application in an operating GIS, reflections and overlapping waves can change both the local voltage-to-current relationship and the accepted zero crossings, so calibration and window selection should be assessed for the installation. Field application also requires evaluation of interference and baseline stability. Denoising is a possible preprocessing step, provided its effects on pulse shape, crossing time, and the double integral are considered.

The contribution of this study is the development and laboratory validation of the VHF-voltage DTIV approach, with emphasis on integration endpoint and threshold selection. In comparison with other methods, FDTD simulates electromagnetic propagation, while the digital model and UHF double-integral relationship provide the charge mapping. HFCT estimates likewise depend on the response and calibration. The other unconventional methods failed to calculate apparent charge quantitively due to non-flat sensor responses.

## 6. Conclusions

This paper proposes a GIS PD charge-estimation method based on the double time integral of VHF voltage. By establishing the equivalent circuit of a capacitively coupled VHF sensor, an approximate proportional relationship between the voltage double integral and charge is obtained under the stated circuit and window assumptions. The feasibility of the method is verified through simulation and experiments.

(1) A tapered GIS simulation and experimental platform is used to examine charge estimation with a capacitively coupled VHF sensor. The study evaluates how the integration endpoint and amplitude qualification threshold affect the proportional relationship between integral output and reference charge.
(2) The simulation results show that DTIV_ZC2_, which uses the second zero-crossing as the integration-window boundary, provides a better aggregate fit for the evaluated library, consistent with reduced finite-window error in the tested responses. Linear-regression analysis shows that the ZC_2_ strategy increases the coefficient of determination from 0.8957 to 0.9585 compared with ZC_1_, while reducing the average relative error to 4.5%.
(3) Both the controllable-waveform injection experiment and the artificial-defect signal experiment demonstrate a linear relationship between the double integral of VHF sensor output and reference charge. The estimated results are generally consistent with the reference results; most errors are within 25%, and some samples exhibit errors below 10%.

## Figures and Tables

**Table 1:** Performance evaluation of DTIV_ZC1_ and DTIV_ZC2_ by linear regression. DTIV_ZC1_ and DTIV_ZC2_, DTIV evaluated at the first and second zero-crossings, respectively; k, fitted DTIV-versus-charge slope; R^2^, coefficient of determination; ε, average relative error.

Method	DTIV_ZC1_			DTIV_ZC2_		
Pulse Type	k (Ω·s)	R^2^	ε	k (Ω·s)	R^2^	ε
Gaussian	1.78 × 10^−9^	0.9045	6.6%	1.92 × 10^−9^	0.9551	4.5%
Rectangular	2.04 × 10^−9^	0.8124	11.2%	2.23 × 10^−9^	0.8275	10.8%
Triangular	1.80 × 10^−9^	0.9067	5.9%	1.95 × 10^−9^	0.9598	4.1%
Exponential 1	1.74 × 10^−9^	0.8995	7.1%	1.92 × 10^−9^	0.9551	5.1%
Exponential 2	1.95 × 10^−9^	0.8512	9.8%	1.92 × 10^−9^	0.9012	5.8%
Artificial	1.90 × 10^−9^	0.9244	5.5%	2.03 × 10^−9^	0.9722	3.8%
Overall	1.85 × 10^−9^	0.8957	7.6%	2.05 × 10^−9^	0.9585	4.5%

**Table 2:** Relative residual distribution of DTIV_ZC1_ and DTIV_ZC2_ linear regression.

Relative Error	DTIV_ZC1_	DTIV_ZC2_
<10%	78.92%	87.95%
10–25%	12.05%	9.64%
>25%	9.03%	2.41%

**Table 3:** Performance evaluation of DTIV_ZC2_ under 1% and 5% peak-threshold conditions by linear regression.

Method	DTIV_ZC2_ (1% Threshold)			DTIV_ZC2_ (5% Threshold)		
Pulse Type	k (Ω·s)	R^2^	ε	k (Ω·s)	R^2^	ε
Gaussian	1.93 × 10^−9^	0.9542	4.2%	1.83 × 10^−9^	0.9518	4.3%
Rectangular	2.15 × 10^−9^	0.8754	7.2%	2.13 × 10^−9^	0.9032	5.7%
Triangular	1.95 × 10^−9^	0.9607	4.6%	1.85 × 10^−9^	0.9621	4.2%
Exponential 1	1.92 × 10^−9^	0.9602	4.6%	1.82 × 10^−9^	0.9573	4.7%
Exponential 2	1.90 × 10^−9^	0.9475	5.4%	1.81 × 10^−9^	0.9545	4.5%
Artificial	2.01 × 10^−9^	0.9704	3.9%	1.93 × 10^−9^	0.9758	3.5%
Overall	2.029 × 10^−9^	0.9608	4.2%	1.933 × 10^−9^	0.9641	3.9%

**Table 4:** Relative residual distribution of DTIV_ZC2_ linear regression under 1% and 5% peak-threshold conditions.

Relative Error	DTIV_ZC2_ (1% Threshold)	DTIV_ZC2_ (5% Threshold)
<10%	87.95%	90.36%
10–25%	8.43%	9.64%
>25%	3.61%	0%

**Table 5:** Parameters of the VHF sensor and measurement chain.

Parameter	Value	Influence on Results
Disk diameter	110 mm	Coupling area and capacitance
Disk thickness	6 mm	Mechanical and parasitic parameters
Installation gap	1 mm	Coupling capacitance and gain
Insulating support	Epoxy resin	Parasitics and insulation boundary
Load resistance	50 Ω	Damping, ringing, and zero crossing
Output port	N-type connector	Impedance continuity and reflection
Inner-conductor radius	90 mm	Propagation mode and impedance
Enclosure radius	207.5 mm	Propagation path and impedance
Internal medium	Air	Discharge and propagation medium
Coaxial cable length	approximately 2 m	Attenuation, delay, and reflection
Sampling rate	≥2.5 GS/s	Zero-crossing detection and accuracy
Cutoff frequency	1.5 GHz	Noise, ringing, and integral tail
Conditioning gain	40 dB	Amplitude calibration and range

## Data Availability

Data is contained within the article.
